# Self-Organization of German-Speaking Displaced People in Austria Between 1945 and 1954: The Zentralberatungsstelle für Volksdeutsche

**DOI:** 10.1177/00220094251401606

**Published:** 2025-12-12

**Authors:** Philipp Strobl

**Affiliations:** Department of Contemporary History, 27258 University of Vienna, Vienna, Austria

**Keywords:** refugee agency, ethnic German refugees, migration regime, postwar Austria

## Abstract

This article examines the collective agency of German-speaking displaced people in Austria between 1945 and 1954, focusing on the Zentralberatungsstelle für Volksdeutsche (Central Advice Bureau for Ethnic Germans) and its role in shaping the country's postwar migration regime and national identity. In the face of exclusion from aid and citizenship, Volksdeutsche refugees organized collectively, leveraging their numbers and invoking the idea of a shared Habsburg heritage to achieve legal equality and integration. The study draws on archival sources to show how refugee lobbying, advocacy and alliance-building resulted in key legislative changes – most notably access to employment and citizenship for approximately 300,000 individuals. Yet the article also reveals the limitations and contradictions of this agency: exclusivist narratives marginalized other groups and internal divisions, as well as state intervention, limited the Zentralberatungsstelle's autonomy. The findings demonstrate that Austria's migration regime and national identity were not simply state-driven, but were actively negotiated by refugees themselves, thereby highlighting the contested nature of postwar integration and nation-building.

By May 1945, Central Europe, and particularly the regions of the former German Reich, was in ruins. Millions were dead. Cities, infrastructure and the economy were destroyed. Nazi tyranny was gone; what was left, however, was great uncertainty. In this rudimentary work-in-progress situation, the country strove to find a new racialized conception of a specifically created ‘Austrian’ identity.^1^ At that time, the dysfunctional Austrian state resembled a giant refugee camp: a population of about 6 million was confronted with a heterogeneous group of about 1.65 million displaced people.^2^ They were roughly divided into Allied DPs, citizens of neutral states, citizens of the Allies’ former enemy states and stateless persons. Among the two latter categories were many German-speaking refugees from Central, Eastern and South-Eastern Europe. Many of them had acquired German citizenship during the National Socialist expansion into their countries. The postwar expulsion of German minorities from Central and Eastern Europe was partly a response to the support for Nazism among these groups before and during World War II. As early as May 1940, British Foreign Office advisors and the Czechoslovak government-in-exile in London had discussed the possibility of resettling ethnic Germans from Eastern Europe. By the war's end, these plans had become more concrete. There was a broad consensus among United Nations members that population transfers would be necessary in the postwar order of Central and Eastern Europe, largely to create stable nation states and to prevent a resurgence of German expansionism.^3^ Outbursts of violence in the immediate postwar years, such as pogroms and ethnic cleansing, and the rapidly emerging Cold War further complicated the situation, bringing new cohorts of mostly German-speaking refugees to Austria.^4^

Non-German-speaking DPs were eligible for support from international associations such as United Nations Relief and Rehabilitation Administration (UNRRA) and later on the IRO (International Refugee Organization).^5^ Classified as ‘ex-enemy’, or ‘non-eligible DPs’, German-speaking displaced people, on the other hand, who were mostly in possession of Nazi-German citizenship due to the National Socialist Germanization programmes in Poland, Czechoslovakia, Hungary, Romania and the Soviet Union,^6^ did not receive such support.^7^ Instead, they were subjected to restrictions on their mobility, faced discrimination in housing and employment, received reduced rations, encountered barriers to admission to Austrian universities and were denied welfare benefits and citizenship rights.^8^ The situation, however, left plenty of room for individual manoeuvre and identities changed frequently according to the refugees’ needs. After all, the categories of ‘volksdeutsch’ and ‘DP’ were bureaucratic constructs and a legal status, therefore, whether somebody fell into one or other group was not set in stone and could often be negotiated.^9^ In this context, the significance of refugee agency – the refugees’ capacity to voice concerns and actively shape their own circumstances – increased as refugees formed self-help organizations and negotiated their positions within the emerging Austrian postwar migration regime.

Between 400,000 and 500,000 German-speaking displaced people are believed to have been stranded in Austria after the war.^10^ They were a very heterogeneous group, ranging from Protestants to Catholics, from social democrats to conservatives, and from people from the industrialized regions of the Sudetenland to traditional farming people of the Danube Swabian region. Most of them came from the areas adjacent to Austria, from Bohemia and Moravia, from Yugoslavia, Hungary and Romania, and thus were born in regions that had been part of the Habsburg Empire until 1918. There was a centuries-old cultural connection between these regions and Austria (with its current borders) and many of the refugees identified themselves with the state and stressed their joint heritage. As historian Tara Zahra pointed out, ‘expellees made legal, moral, and financial claims on the postwar Austrian state by representing themselves as *Altösterreicher*, or as citizens of the former Habsburg empire’.^11^

Regarding German-speaking refugees as ‘stateless foreigners’, however, the Austrian authorities, at first, did not take this historical link into consideration.^12^ In fact, Austria's postwar approach of distancing itself from its society's involvement in the Nazi crimes by making a clear distinction between ‘Austrians’ as ‘victims’ and ‘Germans’ as ‘perpetrators’ supported a further distancing from anything German.^13^ Additionally, Austria sought to avoid conflict with the Soviet Union, which was very reluctant to grant support to German-speaking refugees, who they regarded as potential ‘irredentists’ and ’fascists’, at least during the immediate postwar years.^14^ Citing the Allied Potsdam Agreement, according to which all ‘ethnic Germans’ from Czechoslovakia and Hungary were to be resettled in Germany, both Karl Renner's (Social Democrat) provisional government and Leopold Figl's (Conservative People's Party) successor government made great efforts ‘to get rid of them’ by sending them (or ‘repatriating’, as the official term was) across the northern border into the American or British occupation zones of Germany in 1945 and 1946.^15^ It is believed that around 160,000 German-speaking displaced people from Austria had been relocated to the western zones of Germany.^16^ With the arrival of an additional 124,000 German-speaking refugees after 1946, mainly from Yugoslavia, however, numbers of German-speaking refugees in Austria remained high.^17^

Meanwhile, the situation was somewhat different in the Allied occupation zones in Germany, which received around 12 million German-speaking refugees.^18^ Although many Germans had difficulties accepting the newcomers as full members of society, because ‘they were up to their necks in their own problems and were not eager to take in millions of additional people’,^19^ the German states as successors of the Nazi empire had to bear the burden of the lost war, including full responsibility for taking care of the displaced German population of Eastern and Central Europe.^20^ The expellees were therefore granted legal equality with the local population, but were not allowed to form their own political parties or associations.^21^

Since both food and housing were in short supply in Austria, the war-weary population initially greeted the expellees with hostility^22^: they experienced difficulties in getting support, even for their most urgent supply needs. Unable to receive help from UNRRA or the IRO, many tried to secure support from the Austrian authorities. At least during the first five years after the war, this turned out to be very difficult: hoping that the refugees would ‘simply leave’, the Austrian government usually did not grant them citizenship or political or working rights.^23^ This ‘in-betweenness’ caused them great disadvantage, forcing many to rely upon friends or relatives. Also, since most of them were restricted from entering the job market, they had to make a living at the margins of society by working as agricultural labourers,^24^ or by trading on the black market.^25^ This means that for years, accessing Austrian citizenship was an arbitrary matter of individual initiative for most of the refugees.^26^

This changed during the early 1950s for various reasons. Through strategic and highly successful advocacy, many German-speaking displaced people secured Austrian citizenship by explicitly referencing their historical ties to the Habsburg Empire and their embeddedness within Austrian regional communities. This process not only entailed a redefinition of their own identity – dissociating themselves from the Nazi past – but also contributed to an exclusionary narrative that positioned their non-German-speaking Eastern European neighbours outside of the imagined Austrian future. Central to these efforts were self-support organizations, most notably the Zentralberatungsstelle für Volksdeutsche (Central Advice Bureau for Ethnic Germans), which played a pivotal role in orchestrating collective action and providing crucial support services. Exercising agency across multiple spheres of public and private life, the Zentralberatungsstelle actively sought to reshape public perceptions of German-speaking displaced people, employing a range of strategies to recast them as rightful members of Austrian society and legitimate claimants of citizenship. Thus, the organization not only mediated the integration of the *Volksdeutsche* but also decisively influenced the formation of postwar Austrian identity and memory. Not least due to the lobbying and informational work of the refugees and their representative body, the Zentralberatungsstelle, Austrian authorities gradually began to recognize German-speaking displaced people as economically valuable and culturally and ethnically related to the Austrian majority population.^27^ Furthermore, as it became clear that they would stay in Austria, political parties came to realize the potential of the masses of future voters. A view towards West Germany, where an expellee party was represented in the federal parliament between 1950 and 1958, and was even part of the German government between 1953 and 1955, also showed Austrian officials the high political potential of German-speaking expellees.^28^

From the early 1950s on, there was a shift in public opinion towards thinking of German-speaking refugees as culturally and ethnically related and economically valuable people resulting in a gradual loosening of labour restrictions. Consequently, by 1954 the new *Optionsgesetz* (option law) granted them access to citizenship. Official figures confirm this change in politics: in 1954, 225,000 German-speaking displaced people obtained Austrian citizenship. Overall, an estimated 300,000 of them are believed to have been granted citizenship, during the postwar period.^29^

This article investigates the first decade of the fragmented history of migration in postwar Austria, when a distinctly Austrian national identity, as well as a new migration regime, was formed and negotiated. Following Tara Zahra's observation that *Volksdeutsche*^30^ refugees contributed to the elaboration of this national identity by presenting themselves as *Altösterreicher* with legal, moral and financial claims on the postwar state,^31^ i seeks to analyse how members of this group collectively organized themselves and created new or stressed existing identities referring to the heritage of the by then defunct Austro-Hungarian monarchy and distancing themselves from Nazi Germany and pan-Germanic ideas, as well as from other refugee groups.^32^ German-speaking refugees from Central, Eastern and South-Eastern Europe who came to Austria after World War II had been described as *Volksdeutsche* in the official contemporary terminology in order to separate them from Germans from Germany (so-called *Reichsdeutsche*), as well as from German-speakers from South Tyrol, who were immediately regarded as Austrians.^33^ While the term *Volksdeutsche* was common in contemporary official and public discourses, today it is hardly used, even in the context of the expellee milieu, since refugees aimed at distancing themselves from pan-Germanic ideologies and sought to identify themselves rather with an imagined Habsburg heritage.

In analysing the collective agency of German-speaking displaced persons in Austria in the first postwar decade, this article pays particular attention to the activities of their primary representative organization, the Zentralberatungsstelle für Volksdeutsche. Between 1945 and 1954, this organization emerged as a key actor in negotiations over the legal, social and political status of German-speaking refugees. It established a pervasive presence across multiple spheres of public life in postwar Austria, consistently advocating for recognition of a shared Habsburg heritage grounded in an alleged common culture and ethnicity, as well as underscoring the economic value of the German-speaking displaced people. Adopting an agency-centred perspective is especially productive in analysing migration regimes, which are understood here as structured assemblages of rules, practices, institutions and discourses that regulate and shape cross-border movement and integration.^34^ Migration regimes are, crucially, shaped by actors who – according to their positions within existing power structures – enter the political arena to articulate their interests, values and claims.^35^ Accordingly, this study is particularly concerned with elucidating the strategies and lobbying techniques the Zentralberatungsstelle employed to promote the civic, social and economic rights of German-speaking displaced people within the emerging Austrian migration regime. Through this analysis, the article also seeks to illuminate the broader impact of refugee agency on the formation of a distinctly Austrian migration regime and national identity in the decade following World War II.

The historiography of German-speaking refugees in Austria remains limited, despite the fact that they constituted ‘the most important group of postwar refugees who stayed in Austria’ and played a decisive role in the country's societal and national reconstruction.^36^ Early studies on German-speaking displaced people in Austria emerged primarily from disciplines other than history.^37^ Only 40 years after the first German-speaking refugees had arrived did historians take up the issue.^38^ Existing scholarship on ethnic German refugees in Austria after 1945 has concentrated largely on questions of demography, classification and belonging, on material conditions and institutional responses – including the operation of DP camps, housing provision and labour integration, on their flight trajectories and the relief measures provided, on legal and citizenship issues, and on processes of memory formation and public reception in postwar Austrian society.^39^ However, relatively little is known about their social, economic, or political identification with Austria, or about their capacity for agency within these postwar contexts.^40^

Arriving in Austria during a chaotic time, displaced people encountered a wealth of serious legal, administrative, economic and social problems. In addition, there was little awareness of their plight among wide parts of the population.^41^ Owing to their initially disadvantaged position and the authorities’ reluctance to assume responsibility for their welfare, some German-speaking refugees began to organize autonomously. Through a range of collective initiatives aimed at amplifying their voice, they gradually succeeded in improving their position within the postwar migration regime. Over time, they increasingly portrayed themselves as assets to the Austrian economy and as a population culturally and ethnically aligned with the host society.^42^

The ways in which German-speaking displaced people contributed to the social development and legal negotiation of Austria's postwar migration regime have, to date, largely escaped scholarly attention. In practice, refugees engaged in a wide spectrum of activities, both individually and collectively, aimed at shaping their circumstances and prospects. In academic discourse, such forms of action are commonly conceptualized under the rubric of ‘agency’.

As philosopher Alex Callinicos noted, the concept of agency could be useful to take seriously ‘the apparently platitudinous thought that, in acting, agents exercise powers’.^43^ In this sense, agency may be understood as the ‘ability to influence one's life’, embodied in those ‘actions that are intentionally pursued to exert influence on one's life’.^44^ From this perspective, a central feature of agency is its capacity to enable individuals to participate in their own self-development in accordance with changing historical circumstances.^45^ In doing so, they draw upon various forms of power – some commonly held by all human beings, others structurally determined insofar as they depend on an actor's position within prevailing social structures.^46^

As a central element in migration processes, agency has emerged as a significant topic within historiographical research.^47^ Recent scholarship increasingly demonstrates that the decision to migrate or seek refuge is a highly individual one. In historical contexts of forced migration, research now focuses on those individual actions which reveal how people transcended structural constraints.^48^ Resources and opportunities, as well as collective networks and strategies, proved to be decisive factors for these processes.^49^ The agency of forced migrants is discussed in increasingly reflexive terms in contemporary research, and an emphasis on their self-expression serves to ‘relocate refugees to the centre of historical inquiry’.^50^ Their agency encompasses the capacity to claim, appropriate and utilize categories, institutions and other social structures in order to shape their own lives and challenge institutional arrangements.^51^ This agency is strongly dependent on refugees’ abilities and experiences, such as language acquisition or specialized knowledge of the host society. Analysing these abilities provides a critical perspective on the dynamics between host societies and forced migrants, illustrating, for example, how refugees addressed everyday challenges, including access to the labour market, education, healthcare and housing.

The agency of forced migrants is manifested in various forms. When seeking a framework to structure these, three general modes can be identified. First, there is what social cognitive theory refers to as ‘direct personal agency’.^52^ This term includes all measures individuals took by themselves to influence their environment. In addition, there is so-called ‘proxy agency’, which describes reliance on others to act on one's behalf in order to secure desired outcomes. Finally, there is ‘collective agency’, which is exercised through socially coordinated and interdependent efforts.^53^ This is best understood as the temporary cooperation of individuals who share similar intentions and jointly pursue their aims. It is important though to note that these modes are considered to be analytical categories that help us to simplify our analysis. In practice, multiple actors may collaborate or act in opposition, sometimes simultaneously, within a single organization. The Zentralberatungsstelle, our primary case study, represents one such complex example, where the boundaries between refugee and state organizations became blurred and various power struggles emerged between refugee leaders and government representatives.

At this pont, the text examines the institutional structure of the Zentralberatungsstelle, its personnel composition and its strategic initiatives directed towards Austrian politics at both federal and state levels, highlighting the forms of collective agency that emerged through its and some of its key members’ lobbying activities with official governmental bodies. It also demonstrates that the Zentralberatungsstelle cannot be viewed as a unified actor exercising collective agency according to a predefined or coherent plan. Rather, its existence and activities were characterized by ongoing conflicts among external and internal interest groups. This complexity is illustrated through an analysis of the persistent struggles that took place among the organization's functionaries, between regional branches and headquarters, and between the Zentralberatungsstelle and governmental bodies such as the Ministry of the Interior, which attempted to exert control over its operations.

Due to their precarious circumstances, displaced people were compelled to establish a diverse infrastructure of organizations and associations. These newly formed ‘communities of fate’ constructed identities centred around an imagined Habsburg heritage and joined together – often in ad hoc configurations – to exercise agency within a host society that was slow and initially reluctant to accept them as new citizens. The proliferation of these organizations can be traced primarily to the insufficient support provided to German-speaking refugees, both by the Austrian state and by the international community.

Where it existed, refugee aid happened at the local level. Usually, small-town mayors and priests were the main actors involved, together with the Catholic Church providing logistical aid through their local networks.^54^ In many cases, organizing themselves in ‘self-help groups’ was the refugees’ best option. The countless organizations and associations that mushroomed wherever refugees had arrived enabled them to deal with their most urgent needs, such as the search for missing family members, housing opportunities and food, as well as issues of visas and work permits.^55^

In 1946, efforts by some German-speaking displaced people, who had at this point managed to become politically active, resulted in the formation of a joint representative body aimed at incorporating all of those loosely formed organizations.^56^ This led to the establishment of the Zentralberatungsstelle für Volksdeutsche in Vienna, which managed to become publicly recognized as a major representative body that fulfilled a supporting function for German-speaking displaced people. The organization came into being on 27 April 1946 after acceptance by the Allied Council and the Austrian Minister of the Interior.^57^ Its overall purpose was ‘to look after the ethnic Germans situated in the Federal State of Austria, especially those from the territory of the Austro-Hungarian Monarchy’.^58^ As its organizational statutes reveal, the people involved in this founding process sought to create a connection between the refugees and their joint Austro-Hungarian heritage. To extend its reach into the Austrian states, further regional offices were formed in Salzburg, Upper Austria, Styria, Carinthia, Tyrol and Vorarlberg.^59^

The nature of this organization is complex and, in certain respects, resembles the structural patterns found in expellee associations in West Germany. There, the Bund der Vertriebenen (Federation of Expellees) and its precursor organizations were also closely connected to the state from the very founding of the Federal Republic. They received state funding, participated in legislative processes such as the Federal Expellee Law (1953), and were therefore able to exert direct influence on the formulation of compensation measures, rights and social integration.^60^ In Austria, the Zentralberatungsstelle was established by displaced people to represent themselves, yet its establishment would have been impossible without the support of the Ministry of the Interior, which, according to the sources, regarded it as a semi-official institution that provided a framework to exercise control and influence over a large group of people, who were after a while expected to become Austrian citizens (and voters) at some point. It was, however, an independent association run and led by refugee functionaries and its formation can best be understood in the context of the highly politicized Austrian postwar society. We can see here the interplay of personal, collective and political interests: individuals such as the Danube Swabian (Romanian) Sebastian Werni and the Sudeten German (Czechoslovakian) Rudolf Lukesch used their political affiliations to become central figures in the association's formation.^61^

Exercising agency in postwar Austria was barely possible without the support of one of the major political parties.^62^ Political affiliation was crucial and, according to a 1947 police report, all of the founders of the Zentralberatungsstellen were connected to at least one of the large parties. There was a particularly intense connection between displaced people from Czechoslovakia who had belonged to the Sudeten German Social Democratic Party and the Social Democratic Party in Austria.^63^ Rudolf Lukesch, one of the founders of the Zentralberatungsstelle*,* was a local functionary of the Socialist Party in Czechoslovakia and joined the Austrian Social Democratic Party after his expulsion in 1945.^64^ Sebastian Werni had arrived in Vienna in 1945, where he met Lukesch and other displaced people and developed the plan of forming an ‘ethnic German Committee that worked together with Austrian authorities to facilitate the integration of German-speaking refugees’. First, he contacted the Conservative People's Party (ÖVP), which, however, ‘offered too little possibility to support his cause’.^65^ He went on to establish connections to the Minister of the Interior, Oscar Helmer, and other leading Socialists. Ultimately, he joined the Socialist Party with the main intention of securing more support for his cause.^66^ And, indeed, Helmer, who initially opposed an ‘en-bloc’ naturalization of German-speaking displaced people, supported and accepted the establishment of the Zentralberatungsstelle*,*^67^ of which Lukesch became the chairman.^68^

Although the Zentralberatungsstelle was established by displaced people as an instrument of collective agency, state influence remained omnipresent, and the organization's history was marked by a continual power struggle between its leadership and administration and the Ministry of the Interior. From the outset, the Austrian government sought to extend its influence over the newly formed organization, which was also monitored by the Directorate for Public Security.^69^ The organization's claim to represent approximately 350,000 individuals was particularly significant, making the prospect of influencing its decisions highly attractive to government authorities. Members of the Ministry of the Interior's resettlement office (U 12) were granted the right to attend meetings of the Zentralberatungsstelle, to inspect its correspondence, and even to reject elected members of its leadership. As a result, the state's influence over the organization steadily increased. When, in 1947, members of the Zentralberatungsstelle prepared the election of a central committee, where all German-refugee ethnicities would have been represented, the Minister of the Interior prohibited the endeavour, because ‘it was obviously based on the fundamental idea of turning the Zentralberatungsstelle into a kind of parliamentary representation of the ethnic Germans’. The minister further argued, ‘this is not in line with the tasks of the Zentralberatungsstelle, which must limit itself exclusively to the activities corresponding to its name, and would lead to negative foreign policy entanglements’.^70^

The government was able to exercise a certain degree of control over the Zentralberatungsstelle because it provided financial support. However, even the situation around the headquarters of the organization reflects the struggle for influence between the displaced people and the state. Initially, the Ministry of the Interior had provided the office space for the association. However, according to a report of the organization, it was ‘in disrepair’ and ‘had to be rebuilt’ from the financial means of the organization.^71^ There was additional funding for the regional branches from the Austrian regional states. As a 1950 report shows, the Zentralberatungsstelle in Vienna received federal subsidies, whereas the local branches were usually funded by the regional states as well as membership fees.^72^ Official funding had been a persistent issue in the various negotiations between different actors (the associations, the federal and the state governments). A letter from the Upper Austrian state government to the Zentralberatungsstelle in Linz dated February 1950 provides insight into the associations’ quest for public funding: ‘The application to increase the monthly subsidy from 4000 to 6000 schillings cannot be granted’. The application was denied with the discouraging argument that ‘a random check revealed that some of them have recently been self-sustaining’.^73^

There was also continuous intra-organizational competition for existing funding, as revealed by the minutes of a 1950 meeting of the regional branches, where the Viennese headquarters was asked to come up with its own funding and share their federal subsidies with the regional branches.^74^ A 1950 letter from the Viennese headquarters to the local branches further exemplifies the tense situation, stressing that the Viennese central branch was solely authorized to make decisions that affected all ethnic Germans in Austria and that the regional branches should stop exceeding their remit.^75^

Despite the growing influence of the Austrian government, the Zentralberatungsstelle strove to keep its independence: on various occasions, it opposed decisions by the Ministry of the Interior, or even complained about having too little say in official decisions that affected displaced people. As a letter from 1950 to the Ministry of the Interior reveals, the organization ‘noted with regret that the refugee groups of the ethnic Germans […] are neither represented in the Federal Committee for Refugee Aid nor are they invited to speak about their existential problems’.^76^ The independent appearance vis-à-vis the ministry was possible, as despite its public subsidies, the Zentralberatungsstelle was largely funded by membership fees. Most importantly, however, the organization's claim to represent 350,000 people was a powerful bargaining chip when negotiating with the government:^77^

In the sixth year of its homelessness and legal declassification, the ethnic German element is no longer asking for, but is already being forced to loudly demand, its legal equality with the Austrians. […] To continue to withhold this fundamental right to equality […] would have consequences that no one, least of all the responsible state leadership, would like to be responsible for.^78^

As this press release demonstrates, the members of the Zentralberatungsstelle became increasingly aware of the significance of their large representative body and the authority derived from claiming to represent a substantial population. In this context, it is likely that they drew lessons from developments across the West German border, where expellee associations had leveraged their considerable membership numbers to exert pressure on the government.^79^ In certain instances, as the statement above illustrates, the Zentralberatungsstelle actively employed such claims to exert pressure on the government, utilizing dramatic language and invoking the threat of negative consequences.

In accordance with the available opportunities and the rapidly changing political, social and economic situation, the aims of the Zentralberatungsstelle changed over time. At first, it regarded itself as responsible for the support of all German-speaking displaced people in Austria from Eastern and Southern Europe who aimed to migrate to Germany. This changed in October 1946 when Allied refugee transports ceased. From then on, comprehensive legal equality for all German-speaking displaced people in Austria, as well as welfare issues, became increasingly important, particularly issues relating to family reunification, equal treatment under labour law and the drafting of memoranda.

The Zentralberatungsstellen were well connected and cooperated with a network of proxies, ranging from denominational to welfare organizations.^80^ Within a few years, the association had adapted its aims again, in response to the altered situation, from managing further migration towards negotiating welfare and naturalization issues in Austria. Accordingly, the Zentralberatungsstelle, as an instrument of collective agency of forced migrants, increasingly became a key player within the negotiations of the Austrian migration regime: it established close ties not only to political parties but also to the government, local authorities, the Catholic Church and different denominational aid organizations, such as the Quakers or the Church of Brethren, as well as professional interest groups such as the chambers of commerce or the Gewerkschaftsbund (Federation of Trade Unions).^81^ As its 1949 report shows, the Zentralberatungsstelle in Vienna had intervened with most of the governmental institutions at various levels (Ministry of the Interior, Ministry for Social Administration, Ministry for Agriculture, Ministry for Finance, Ministry for Trade and Reconstruction, Ministry for Asset Protection, Ministry of Economic Planning, Ministry of Justice, the Federal Chancellery, provincial governments, employment offices, police directorates, the IRO and Caritas, as well as the Allied Administration).^82^ Its leadership used its political affiliations to lobby governmental agencies and to pressurize the governments and politicians by referring to the collective influence of the organization. A 1950 telegram sent by the board of the organization to the Austrian Chancellor, Leopold Figl, offers insight into the self-confidence the organization sought to display when sending best regards ‘in the name of the 350,000 ethnic Germans’, immediately referring to their ‘unbearable situation’, and demanding that the federal government immediately begin to solve the ‘ethnic German question’.^83^

This part of the paper will outline several concrete initiatives pursued by the Zentralberatungsstelle during the 1940s and early 1950s to influence negotiations concerning the legal equalization of displaced people in Austria. These cases serve to specify the notion of collective agency by highlighting the concrete actions undertaken by the organization and its leadership to bring refugee concerns onto public and political agendas. One of the most pressing issues for many forced migrants in Austria was their exclusion from the national labour market. According to the regulations in place at the time, employment offices were only authorized to place Austrian nationals or individuals in possession of a *Gleichstellungsschein* (certificate of legal equality). In practice, however, non-Austrian refugees were almost never granted such certificates and were therefore systematically prevented from accessing formal employment.^84^ Only the low-income fields of agriculture and construction were open to them. Even if someone had found a job, employers were not allowed to hire them without a *Gleichstellungsschein*. Strong opposition towards the professional and legal integration of forced migrants came from both sides: the powerful labour unions (Gewerkschaftsbund) as well as professional representations. German-speaking displaced people in Austria thus found themselves in a difficult position, in contrast to Germany, where the expellees were granted legal equality with the native German population.^85^

The case of approximately 60 German-speaking rail workers and their families from Romania gives a deep exemplary insight into the ‘in-betweenness’ of many displaced people in Austria during the late 1940s. They were resettled into Nazi Germany in 1940 and consequently employed by the German Reichsbahn. After the end of the war, they found themselves in the Republic of Austria. They were not allowed to move into the western zones of Germany where they could have easily applied for citizenship. Like hundreds of thousands of other German speakers, they were considered aliens in Austria. Unable to find employment, they were crammed into a refugee camp in Salzburg. After living there for four years, without any chance of finding regular employment, they asked for the support of the Zentralberatungsstelle, which in August 1949 tried to negotiate their emigration to Germany and their transfer to the German railways. However, the efforts were unsuccessful, as a reply from the German Federal Railway indicates: ‘Unfortunately, it is not possible for us to employ the railway workers of ethnic German origin from Romania in our area, because our need for labour of all kinds is covered for a long time’.^86^ It is not known what happened to those 60 people, as the file from the Zentralberatungsstelle ends at that point, however, it is likely that their situation continued for some more years, as was the case with most of the other German-speaking refugee aliens who had no access to citizenship in Austria during the postwar years. The Zentralberatungsstelle continued its intense lobbying work, as its annual reports reveal. In Styria, the Zentralberatungsstelle had collected letters from employers of *Volksdeutsche* and sent them to the Ministry of the Interior, describing them as valuable assets for the Austrian economy. One of them even noted that ‘a comparison with other foreign workers is not even possible because the others could never equal the *Volksdeutsche*.’^87^ It was not until 1951 that the Federal Ministry for Social Administration enacted more generous labour regulations against the resistance of the Austrian labour unions, allowing German-speaking displaced people to receive a *Gleichstellungsschein* and thus be able to find regular employment.^88^ One year later, in December 1952, the government granted equal rights to German-speaking refugees who sought to work as self-employed businessmen.^89^

An important field of collective agency of the Zentralberatungsstelle was the lobbying work with Austrian professional representations and labour unions. A good example of resistance against a professional integration of displaced people and the subsequent agency enacted against it was given by the federal Chamber of Lawyers (Rechtsanwaltskammer). It expressed ‘fundamental concerns’ over a full integration of German-speaking refugee lawyers in Austria.^90^ Reasons for the resistance were ‘fear of additional competition’, as well as the alleged ‘incompatibility of foreign degrees’.^91^ The Zentralberatungsstelle responded in petitions and letters. As it wrote in 1949, the fear of competition ‘is all the more deplorable because the number of ethnic German lawyers is so small that there can be no question of professional competition’.^92^ According to a letter to the Austrian Minister of Justice, there were only 10 German-speaking lawyers in Upper Austria who were in ‘material and emotional distress’ and required the state to recognize their ability to exercise their profession.^93^

Displaced German-speaking doctors found themselves in a similar position. As an undated memorandum shows, there were only 120 such doctors in Austria. Of those, 50 had even studied at an Austrian university, 38 had studied at the ‘German Karl's University in Prague’ and 13 had received their degree from a German university. The memorandum further noted that, on average, those 120 doctors had practiced medicine for 17 years, ‘which would make a change in their profession virtually impossible’.^94^ Despite their small number, physicians seem to have been well represented in the Zentralberatungsstelle. In 1949, they managed to form their own representation, the ‘Provisorische Ärztevertretung’ and hold annual ‘ethnic-German medical representatives conferences’.^95^ According to a letter from 1949, the role of this elected representative body would be ‘to represent the interests of the profession in the future solution of the national German problem, to draw up appropriate proposals and to liaise with all relevant bodies today’.^96^ In several letters to the government, the physicians’ representation demanded that ‘all ethnic German doctors be authorized to care for their compatriots and to practice medicine in Austria and be treated equally to Austrian doctors in terms of their rights and duties’.^97^ Representatives of the displaced German-speaking physicians organized within the framework of the Zentralberatungsstelle also directly lobbied the Ärztekammer (Austrian Medical Association). Here, again, they applied pressure by referring to the apparent lack of health care for refugees as a dangerous locus of disease for Europe, which had to be resolved immediately to prevent a catastrophe.^98^ In September 1949, representatives of the refugee physicians attended the Austrian Ärztekongress, an annual gathering of high-ranking Austrian physicians. They met the president of the Austrian Medical Association and gave him a memorandum about their difficult situation.^99^ The physicians’ representation, however, not only lobbied for German-speaking refugee doctors but also used their collective agency to organize free-of-charge health services for other German-speaking displaced people in Austria: together with proxies – in particular the Protestant Brethren Service Commission – they organized a specialist medical advice centre in the city of Linz, where eight German-speaking refugee doctors provided medical advice to refugees.^100^ Even after lawyers and physicians had grouped together to exercise agency in the framework of the Zentralberatungsstelle, it would take until 1952 for laws to be enacted which would allow them to practice their professions.^101^

One of the foremost aims of the Zentralberatungsstellen was the naturalization and thus equalization of their members.^102^ In the context of postwar reconstruction and recovery, refugees were not very prominent on public agendas. Additionally, awareness of the difficult refugee situation was also very low.^103^ As the Zentralberatungsstelle and its regional branches increased pressure, they managed to thematize the situation in the public discourse. As a 1986 study on the political integration of German-speaking refugees claims, ‘it was mainly because of the work of the Zentralberatungsstellen that an atmosphere of sympathy for the situation of the ethnic Germans was created with the authorities and the government’.^104^ Of course, a variety of additional factors influenced the process of equalizing the rights of German-speaking displaced people. From around 1949 onwards, the attitudes of political parties towards German-speaking displaced people began to shift. As a sufficient number of German-speaking displaced people had become Austrian citizens and could participate in elections, campaigning for refugee votes had started. This change coincided with developments in West Germany, where authorities had adopted a more supportive policy towards expellees by 1948. A series of legislative measures aimed at improving the situation of expellees were introduced, including the *Soforthilfegesetz* (Immediate Relief Act) of 1949 and the *Lastenausgleichsgesetz* (Equalization of Burdens Act) of 1952.^105^

An internal 1948 ÖVP note gives an early insight into the political debate around potential refugee votes. The letter – classified as ‘strictly confidential’ – warned against ‘Social Democratic Party of Austria (SPÖ) manoeuvres for ethnic Germans’. It further mentioned that the SPÖ Minister of the Interior would do anything to win ethnic Germans for his cause.^106^ Mistrust was common on both sides of the political spectrum and the SPÖ leadership also feared that the ÖVP, which had nominated a refugee candidate in 1949 for a seat in parliament, was ahead in the race for refugee votes. After the elections in autumn 1949, the issue of integrating expellees into the voting blocs of the ÖVP and the SPÖ became a real concern. One decisive factor for this new awareness was the formation of a new party, the Verband der Unabhängigen (Union of Independents), which sought to appeal not only to former Nazis, who had not been allowed to vote so far, but also to other social groups that had become politically homeless, including German-speaking displaced people.^107^ In contrast to the situation in Germany, where expellees established their own political party – the Bund der Heimatvertriebenen und Entrechteten (BHE) (Association/Federation of Expellees and Disenfranchised Persons), later renamed Gesamtdeutscher Block/BHE (GB/BHE) (All-German Bloc/Federation of Expellees and Disenfranchised Persons) – which was represented in the federal parliament (Bundestag) from 1950 to 1957 and even participated as a coalition partner in the federal government from 1953 to 1955,^108^ German-speaking displaced people in Austria were never able to form a comparable party-political representation.

As a result of this politicization process, many prominent members of the Zentralberatungsstelle became active as functionaries within established political parties, most notably the conservative People's Party or the Social Democratic Party. However, this development did not represent a coordinated initiative by a unified collective of refugee representatives. Rather, it was driven by highly individualized and often competing efforts, reflecting divergent personal ambitions and strategies among the organization's leadership. The Sudeten German (Czechoslovakian) refugee Erwin Machunze, who had been involved in the establishment of the Zentralberatungsstelle, became the first member of parliament with a German-refugee background for the People's Party in 1949. In this function, he lobbied for equalization and, in January 1951, parliament enacted laws entitling German-speaking refugees to unemployment aid.^109^ As a member of the Social Democratic Party, Sebastian Werni had lobbied the Social Democratic Gewerkschaftsbund and had finally secured the organization's approval for an equal legal status under the labour laws for most of the refugees. A letter, written by Johann Böhm, the head of the Gewerkschaftsbund, gives a profound insight into Werni's groundwork:

The interest group of ethnic German expellees in Austria has requested that the Austrian Labour Unions should agree to the complete equality of ethnic German workers and employees with Austrian employees under labour law and the abolition of the previously required exemption certificates. I am now in a position to inform you, Mr Federal Minister, that the Federation of Labour Unions considers the wishes of the ethnic Germans to be justified and requests that you initiate their equalization as soon as possible.^110^

In 1951, only one month later, the Ministry of Social Administration issued a decree which included German-speaking displaced people in employment law. Further legal improvements resulted from continuing negotiations between the Zentralberatungsstelle, their proxies and the government: in 1952, German-speaking forced migrants were included in the legal provisions for maternity protection, war victims’ pensions and disability benefits.^111^ As mentioned previously, from then on it was possible to achieve equal treatment for refugee doctors, dentists, pharmacists, midwives, craftsmen, and notaries and lawyers, although here, too, the negative attitude of several interest groups often complicated this process.^112^ The year 1953 brought equal rights for German-speaking war veterans, widows and orphans.

Discussions about discrimination against the refugees continued until July 1954, when the parliament passed a law allowing ‘persons of German language who are stateless or whose nationality is unclear (ethnic Germans)’ to acquire citizenship by expressing ‘that they wish to belong to the Republic of Austria as loyal citizens’.^113^ With this law, most of the refugees’ legal demands had been fulfilled. At this point, German-speaking refugees had managed to become a ‘visible presence’ in the country, as historian Gaëlle Fisher has put it.^114^ Gradually, the Zentralberatungsstelle's importance as the central instrument of collective refugee agency decreased and other organizations gained importance, such as the more regionally focused Landsmannschaften (homeland associations) which represented the refugees according to their regions of origin. From 1954, their federal body, the Verband der Volksdeutschen Landsmannschaften in Österreich channelled most of the agendas to ensure civic and social identification of German-speaking refugees.^115^ By 1955, the Viennese headquarters of the Zentralberatungsstelle had been dissolved and there were only four regional branches left, whose main services were described as liaison between the individual refugees, the government and voluntary organizations.^116^

The first postwar decade in Austria was shaped by the challenge of integrating a sizable, heterogeneous group of German-speaking displaced people as Austrian society rebuilt itself both materially and symbolically. While these refugees initially faced exclusion and legal uncertainty, the collective agency they exercised – primarily through the Zentralberatungsstelle für Volksdeutsche – became a decisive and multifaceted force in shaping both their own futures and Austria's developing migration regime.

Understanding how forced migrants negotiate their position within host societies is central to global migration studies. The agency of the Zentralberatungsstelle illustrates these complex processes: its establishment and function were marked not only by diversity of identity and loyalty within the refugee group but also by a politicized postwar Austria, in which most civil society organizations required major party affiliation. The very process of forging and maintaining the Zentralberatungsstelle was characterized by ongoing struggles – between the organization and the government, between headquarters and regional branches, and among the interests within its membership.

Collective agency here was neither stable nor unified; it arose through negotiation, adaptation and, at times, conflict. The Zentralberatungsstelle's efforts were continually challenged by internal divisions and state attempts to assert control. Nevertheless, its strategy of pragmatic engagement – leveraging lobbying, public advocacy and shifting political alliances – over time supported or triggered key legislative achievements, such as access to the labour market and citizenship and professional recognition. The leadership's readiness to change affiliations based on political opportunity further illustrates the situational and transactional character of agency in this context.

The refugees’ in-between legal and social status both compelled and made possible a form of collective self-representation that could articulate their interests. The Zentralberatungsstelle not only claimed to represent up to 350,000 displaced people as a source of bargaining power but actively built networks with other groups to support its aims. It also served as a platform for the legal and social identification of German-speaking refugees, contributing lasting frameworks for their equalization and integration.

However, the impact of the Zentralberatungsstelle's agency was not evenly distributed. Its emphasis on a shared Habsburg heritage fostered exclusivist narratives, leaving other refugee groups marginalized. Internal divisions and selective advocacy illustrate the contingent character of collective agency, while shifting relations with authorities underscore its negotiated nature.

Analytically, this case emphasizes that postwar migration regimes and national identification were not simply imposed but were the result of contestation and adaptation by marginalized groups themselves. The experience of the Zentralberatungsstelle exemplifies both the potential and limits of collective refugee self-organization in effecting change, as well as the complex power relations in which such organizations operate.

## Notes

1 T. Zahra, ‘“Prisoners of the postwar”: Expellees, displaced persons, and Jews in Austria after World War II’, *Austrian History Yearbook* 41 (2010), 191–215, 193.

2 Österreichischer Nationalrat, X. Gesetzgebungsperiode, III-46 d.B., Bericht des Bundesministers für Inneres über die Flüchtlingssituation in den Jahren 1945–1961 und über die Auflösung der Altflüchtlingslager in Österreich.

3 I. Nachum and S. Schaefer, ‘The semantics of political integration: Public debates about the term “expellees” in post-war western Germany’, *Contemporary European History* 27, 1 (2018), 42–58, 46.

4 P. Strobl and N. Hagen, ‘New perspectives on displaced persons (DPs) in Austria’, *zeitgeschichte* 47, 2 (2020), 165–80, 167.

5 P.-P. Volf, ‘Der Politische Flüchtling als Symbol der Zweiten Republik. Zur Asyl- und Flüchtlingspolitikseit 1945’, *zeitgeschichte* 11–12 (1995), 415–35, 421.

6 D. Bergen, *The Holocaust: A New History* (Barnsley 2008)

7 Zahra, ‘Prisoners of the Postwar‘, 195; G. Fisher, ‘“Ethnic Germans”, “Expellees” or “Old Austrians”: Identity Narratives and Memory Politics of Germans from Bukovina in Austria since 1945‘, in M. Röger and A. Weidle (eds) *Bukowina-Deutsche: Erfindungen, Erfahrungen und Erzählungen einer (imaginierten) Gemeinschaft seit 1775* (Berlin 2020), 152.

8 Zahra, ‘Prisoners of the Postwar’, 195.

9 J. Panagiotidis, ‘Not the concern of the organization?’ The IRO and the overseas resettlement of ethnic Germans from eastern Europe after the Second World War’, *Historical Social Research*, 45, 4 (2020), 173–202, 175.

10 The statistical data differ greatly and can only be regarded as estimates until the first official census in 1948, cf. T. Radspieler, *The Ethnic German Refugee in Austria, 1945 to 1954* (The Hague 1955), 37–43.

11 Zahra, ‘Prisoners of the Postwar’, 194.

12 Fisher, ‘Ethnic Germans’, 152; see also M. Dejnega, ‘Zwischen Nachkriegschaos und Kaltem Krieg: Alliierte Flüchtlingspolitik und die Versorgungssituation von “volksdeutschen” Ex-Enemy DPs’, in C. Defrance, J. Denis and J. Maspero (eds) *Personnes Deplacées et guerre froide en Allemange occupée* (Frankfurt 2015), 213–29.

13 Fisher, ‘Ethnic Germans’, 153; O Rathkolb, *Die paradoxe Republik: Österreich 1945 bis 2005* (Vienna 2025), 38.

14 B. Perching, ‘Migration, Integration und Staatsbürgerschaft – was taugen die Begriffe noch?’, in H. Langthaler (ed.) *Integration in Österreich: Sozialwissenschaftliche Befunde* (Innsbruck 2010), 13–33; M. Stickler, ‘Vertriebenenintegration in Österreich und Deutschland – ein Vergleich’, in M. Gehler and I. Böhler (eds) *Verschiedene europäische Wege im Vergleich: Österreich und die Bundesrepublik Deutschland 1945/49 bis zur Gegenwart* (Innsbruck 2007), 416–33, 424.

15 Fisher, ‘Ethnic Germans’, 152–3.

16 Stickler, ‘Vertriebenenintegration’, 423; P. Schreiner, ‘Aspekte einer Ablehnu,ng: Österreich und die sudetendeutschen Vertriebenen 1945–1948’, MA thesis, University of Vienna (2007), 12.

17 Stickler, ‘Vertriebenenintegration’, 423.

18 P. Ther, ‘The Integration of expellees in Germany and Poland after World War II: A historical reassessment’, *Slavic Review* 55 (1996), 779–805, 781.

19 Nachum and Schaefer, ‘The semantics of political integration’, 42–58, 52.

20 A. Kossert, *Kalte Heimat: Die Geschichte der deutschen Vertriebenen nach 1945* (Munich 2008), 87.

21 Kossert, *Kalte Heimat*, 88.

22 Zahra, ‘Prisoners of the Postwar’, 194.

23 J.A. Seale, ‘Between Austria and Germany, Heimat and Zuhause: German-speaking Refugees and the Politics of Memory in Austria’, PhD thesis, Georgetown University (2020), 33.

24 This was the only kind of work generally allowed for them due to a striking lack of labour force.

25 Perching, ‘Migration, Integration’, 95.

26 Fisher, ‘Ethnic Germans’, 167.

27 Zahra, ‘Prisoners of Postwar’, 215.

28 S. Wolff, ‘German expellee organizations between “homeland” and “at home”: A case study of the politics of belonging’, *Refuge: Canada's Journal on Refugees* 20, 1 (2001), 52–64, 55.

29 Ibid., 154, Zahra, ‘Prisoners of the Postwar’, 209.

30 The term originated in the early 1900s and was used for Germans who lived outside of the area of the German states, particularly during the National Socialist period, cf. S. Müller, *Sprachwörterbücher im Nationalsozialismus: Die ideologische Beeinflussung von Duden, Sprach-Brockhaus und anderen Nachschlagewerken während des* ‘*Dritten Reichs’* (Stuttgart 1994), 144ff.

31 Zahra, ‘Prisoners of the Postwar’, 194.

32 N. Perzi, ‘Flüchtlinge und Vertriebene in der Republik Österreich’, in *Online-Lexikon zur Kultur und Geschichte der Deutschen im östlichen Europa*, https://ome-lexikon.uni-oldenburg.de/p32875 (accessed 16 April 2015).

33 Radspieler, ‘The Ethnic German Refugee’, 5–6.

34 F. Düvell, *‘*The power of migration regimes’ in F. Düvell and S. Makaryan (eds) *Migration Regimes: A Global Perspective* (Amsterdam 2013).

35 C. Rass and F. Wolff, ‘What is a migration regime? Genealogical approach and methodological proposal’, in A. Pott, C. Rass and F. Wolff (eds) *Was ist ein Migrationsregime? What Is a Migration Regime?* (Wiesbaden 2018), 19–64, 45.

36 M. Graf, ‘Austria as a Cold War refuge: Reassessing the historiography’, *Zeitschrift für Ostmitteleuropa-Forschung* 71 (2022), 629.

37 Radspieler, ‘The ethnic German refugee’; A. Bohrmann, ‘Die Volksdeutschen Vertriebenen in Österreich’, *Zeitschrift für Ostmitteleuropa-Forschung* 10 (1961), 452–61; B. Scheuringer, *30 Jahre danach: Die Eingliederung der volksdeutschen Flüchtlinge und Vertriebenen in Österreich* (Vienna 1983); E. Machunze, *Vom Rechtlosen zum Gleichberechtigten: Die Flüchtlings- und Vertriebenenfrage im Wiener Parlament,* vol. 1, *Die V. Gesetzgebungsperiode (1945*–*1949)* (Salzburg 1974); E. Machunze, *Vom Rechtlosen zum Gleichberechtigten: Die Flüchtlings- und Vertriebenenfrage im Wiener Parlament*, vol. 2, *Die VI. Gesetzgebungsperiode (1949*–*1952)* (Salzburg 1976).

38 G. Stieber, *Nachkriegsflüchtlinge in Kärnten und der Steiermark* (Graz 1997); N. Perzi, ‘Aufnahme und Abschub: Die Sudentendeutschen in Niederösterreich 1945/46’, in *Jahrbuch für Landeskunde von Niederösterreich* (2016), 135–233; G. Stieber, *Flüchtlingsnot und Flüchtlingshilfe in Kärnten nach 1945* (Klagenfurt 1999); E.Salvador-Wagner, *Heimat auf Zeit: Das volksdeutsche Flüchtlingslager Haiming 1946–1960* (Innsbruck 1996); Stickler, ‘Vertriebenenintegration’, 416–35.

39 See for example: E. Stanek, *Verfolgt, verjagt, vertrieben: Flüchtlinge in Österreich von 1945-1984* (Vienna 1984); G. Steinacher, *Flucht vor Nürnberg? NS-Täter in Österreich* (Innsbruck 2001), 35; D. Bacher, ‘Verschleppt in eine neue Heimat. Die Integration ehemaliger ziviler Zwangsarbeiter in Österreich nach 1945’, in S. Karner and B. Stelzl-Marx (eds) *Migration: Flucht, Vertreibung, Integaration* (Graz 2019), 77–90; H. Uhl, ‘Gedächtnispolitik in Österreich nach 1945’, *Zeitgeschichte* 37/5 (2010), 243–261.

40 Although in recent times there has been a slightly increased scholarly interest in the issue, see for example: O. Rathkolb, ‘Verdrängung und Instrumentalisierung: Die Vertreibung der Sudetendeutschen und ihre verspätete Rezeption in Österreich’, in B. Coudenhove-Kalergi and O. Rathkolb (eds) *Die Beneš-Dekrete* (Vienna 2012), 138–51; M. Dejnega, ‘“Heimat” im Gepäck? Die Bedeutung der Migrationserfahrung in Lebensgeschichten “deutscher Vertriebener” in Österreich’, PhD thesis, University of Bielefeld (2018), available at https://pub.uni-bielefeld.de/record/2942631 (accessed 25 October 2024); Fisher, ‘Ethnic Germans’; Zahra, ‘Prisoners of the Postwar’.

41 E. Landsmann, ‘Die politische Integration der Volksdeutschen Flüchtlinge und Vertriebenen in die österreichische Gesellschaft im Zeitraum 1945–1955’, MA-thesis, University of Vienna (1986), 69.

42 Zahra, ‘Prisoners of the Postwar’, 202.

43 A. Callinicos, *Making History : Agency, Structure, and Change in Social Theory* (Leiden 2004), xx.

44 M.H. Kristiansen, ‘Agency as an Empirical Concept: An assessment of Theory and Operationalization’, *NIDI Working Paper*, 9 (2014), 3; see also A. Bandura, ‘Toward a Psychology of Human Agency’, *Perspectives on Psychological Science,* 1 (2006), 164–80; S. Hitlin and G. Elder, ‘Time, Self, and the Curiously Abstract Concept of Agency’, *Sociological Theory,* 25 (2007), 137–60; M. Emirbayer and A. Mische, ‘What Is Agency?’, *American Journal of Sociology,* 103 (1998), 962–1023.

45 Bandura, ‘Social Cognitive Theory’, 2.

46 Kristiansen, ‘Agency’, 6.

47 S. Huhn, ‘Negotiating Forced Migration in the IRO's “Care and Maintenance” (CM/1)Files. One Setting, Three Underlying Aims, (at Least) Four Actors, and Multiple Forms of Human Agency’, *IMIS Working Papers*, 12 (2021); C. Rass and I. Tames, ‘Negotiating the aftermath of forced migration: A view from the intersection of war and migration studies in the digital age’, *Historical Social Research*, 45 (2020): 7–44.

48 O. Bakewell, ‘Some reflections on structure and agency in migration theory’, *Journal of Ethnic and Migration Studies* 36/10 (2010), 1689–1708.

49 P. Strobl, “Displaced Knowledge: Austrian Refugees from National Socialism in Australia (Leiden: Brill, 2025), 331f; P. Strobl, ‘Social networks of Austrian refugee-migrants from the Anschluss in Australia — An analysis of meaning structures’, *Journal of Migration History* 5, 1 (2019), 53–79.

50 P. Gatrell, ‘Reckoning with refugeedom: Historical perspectives’, in K. Jacobsen and N. Majidi (eds) *Handbook on Forced Migration* (Cheltenham 2023), 142–48; P. Gatrell et al., *Refugee Voices in Modern Global History: Reckoning with Freedom* (Oxford 2025).

51 Cf. Wehner and Rass, ‘Disputed (Non-)Belonging’, in this special issue; H. Safouane, A. Jünemann and S. Göttsche, ‘Migrants’ agency: A re-articulation beyond emancipation and resistance’, *New Political Science* 42 (2020), 197–217, 216.

52 Bandura, ‘Social Cognitive Theory’, 1.

53 Ibid.

54 P. Aro, ‘After the expulsion: Post-war Austria and “Ethnic German” refugees’, https://www.academia.edu/40858199/After_the_expulsion_Post_war_Austria_and_Ethnic_German_refugees (accessed 25 October 2024).

55 Landsmann, ‘Politische Integration‘, 79.

56 Ibid., 94.

57 Ibid.

58 BMfInneres, Zahl 61.262-12U/46 ‘Zentralberatungsstelle der Volksdeutschen – Organisationsstatut’, Wien am 27.04.1946, an die Zentralberatungsstelle der Volksdeutschen.

59 Wegwarte, Nr. 6, 03.02.1951, 1; Zahra, ‘Prisoners of the Postwar’, 202.

60 Wolff, ‘German Expellee Organizations’; Kossert, *Kalte Heimat*, 146.

61 ‘Volksdeutsche Umtriebe in Österreich’ (28 June 1947), 28.06.1947, 77.518-2/47, 27.431-2/47, Direktion für öffentliche Sicherheit, Bundesministerium für Inneres, Archiv der Republik, Österreichisches Staatsarchiv, Vienna; Sudetenpost 17, 16.September 1961, 5.

62 P. Strobl, ‘Österreich in der Zweiten Republik: Ein kurzer historischer Abschnitt’, in P Strobl (ed.) *Österreich in der Zweiten Republik: Ein Land im Wandel* (Hamburg 2014), 35–52, 39.

63 S. Werni, *Neue Heimat in Österreich: Gelöste und Nichtgelöste Probleme Auskünfte und Hinweise* (Vienna 1970), 13.

64 ‘Volksdeutsche Umtriebe in Österreich’ (28 June 1947), 28.06.1947, 77.518-2/47, 27.431-2/47, Direktion für öffentliche Sicherheit, Bundesministerium für Inneres, Archiv der Republik, Österreichisches Staatsarchiv, Vienna; Sudetenpost 11, 10. Juni 1967, 6.

65 J. Krumpholz, ‘Nachruf an Sebastian Werni’. Available at https://kolut.wordpress.com/2015/02/15/nachruf-an-sebastian-werni/ (accessed 5 November 2024).

66 Ibid.

67 Cf. *Neues Österreich*, 30 November 1946, 2.

68 ‘Volksdeutsche Umtriebe in Österreich’ (28 June 1947), 28.06.1947, 77.518-2/47, 27.431-2/47, Direktion für öffentliche Sicherheit, Bundesministerium für Inneres, Archiv der Republik, Österreichisches Staatsarchiv, Vienna.

69 ‘Volksdeutsche Umtriebe in Österreich’ (28 June 1947), 28.06.1947, 77.518-2/47, 27.431-2/47, Direktion für öffentliche Sicherheit, Bundesministerium für Inneres, Archiv der Republik, Österreichisches Staatsarchiv, Vienna.

70 Letter from the Ministry of the Interior to the Zentralberatungsstelle der Volksdeutschen, 30.04.1947, cited in Landsmann, ‘Politische Integration’, appendix 2.

71 Interessensgemeinschaft Volksdeutscher Heimatvertriebener (eds), *Neue Heimat Österreich: Gelöste und nicht gelöste Probleme, Auskünfte und Hinweise* (Vienna Jugend und Volk, 1970), 10; VLÖ Archiv, Box 10, Instandsetzung des Raumes: Metternichgasse 3/II/133, 13.12.1949.

72 OöLA, ZBST, Ordner 111-113, Sch.40, Verhandlungsschrift.

73 OöLA, ZBST, Ordner 162-168, Sch. 59, Finanzierung der Zentralberatungsstelle der Volksdeutschen, author's translation. Original text: ‘Eine stichprobenartige Überprüfung der ZB-Nebenstellen ergab ferner, dass einzelne davon sich in letzter Zeit selbst erhielten’.

74 OöLA, ZBST, Ordner 111-113, Sch.40, Verhandlungsschrift.

75 ‘Volksdeutsche Umtriebe in Österreich’ (28 June 1947), 28.06.1947, 77.518-2/47, 27.431-2/47, Direktion für öffentliche Sicherheit, Bundesministerium für Inneres, Archiv der Republik, Österreichisches Staatsarchiv, Vienna.

76 VLÖ Archiv, Box 10, An das Bundeskommitee für Flüchtlingshilfe, author's translation. Original text: ‘das Kommitee hat mit Bedauern festgestellt, dass die […] Flüchtlingsgruppen der Volksdeutschen im Bundeskomitee für Flüchtlingshilfe weder vertreten sind, noch zu den Besprechungen über ihre gewichtigen Daseinsprobleme eingeladen werden’.

77 OöLA, ZBST, Ordner 111–113, Sch.40, Pressecommunique.

78 Ibid., author's translation: Original text: ‘Was das volksdeutsche Element im sechsten Jahr seiner Heimatlosigkeit und rechtlicher Deklassierung nun nicht mehr bittet, sondern schon laut zu fordern gezwungen wird, ist seine rechtliche Gleichstellung mit den Österreichern. […] Dieses Grundrecht auf Gleichstellung einer Menschengruppe […] weiter noch vorzuenthalten, würde Folgen zeitigen, die niemand, am wenigsten die verantwortliche Staatsführung verantworten könnte’.

79 Kossert, *Kalte Heimat*, 146–147.

80 Cf. Tätigkeitsbericht der ZB Wien, 08.11.1946, in Landsmann, ‘Politische Integration’, 97.

81 Landsmann, ‘Politische Integration’, 98.

82 VLÖ Archiv, Box 10, Tätigkeitsbericht vom 31.12.1949.

83 OöLA, ZBST, Ordner 111–113, Sch.40, Pressecommunique.

84 Machunze, *Vom Rechtlosen*, 13.

85 Kossert, *Kalte Heimat*, 88.

86 VLÖ Archiv, Box 10, Reichsbahndirektion Stuttgart, author's translation. Original text: ‘Es ist uns leider nicht möglich, die in Frage stehenden Eisenbahner volksdeutscher Herkunft aus Rumänien in unserem Bereich zu beschäftigen, weil unser Bedarf an Arbeitskräften aller Art auf lange Zeit gedeckt ist’.

87 Zahra, ‘Prisoners of the Postwar’, 202.

88 Machunze, *Vom Rechtlosen*, 13.

89 Donauschwäbische Arbeitsgemeinschaft in Österreich (ed.), *60 Jahre 1949–2009* (Vienna 2009), 60.

90 215 der Beilagen zu den stenographischen Protokollen des Nationalrates VII.GP., Bericht und Antrag des Justizausschusses, https://www.google.com/url?sa=t&rct=j&q=&esrc=s&source=web&cd=&ved=2ahUKEwiBmLHf-PWCAxVrhv0HHbpvDrUQFnoECBcQAQ&url=https%3A%2F%2Fwww.parlament.gv.at%2Fdokument%2FVII%2FI%2F215%2Fimfname_333818.pdf&usg=AOvVaw3_xu8XVEeKjv4GeOLJIfQp&opi=89978449 (accessed 28 October 2024).

91 OöLA (Oberösterreichisches Landesarchiv), ZBST, Ordner Nr. 11–113, Sch.40, Volksdeutsche Rechstanwälte.

92 OöLA, ZBST, Ordner Nr. 11–113, Sch.40, Volksdeutsche Rechstanwälte.

93 OöLA, ZBST, Ordner 162–168, Sch.50, Volksdeutsche Rechtsanwälte.

94 OöLA, ZBST, Ordner 162–168, Sch.50, Denkschrift zur Lösung der volksdeutschen Ärztefrage.

95 VLÖ Archiv, Box 10, Betrifft: Ärztevertretertagung.

96 OöLA, ZBST, Ordner 162–168, Sch.50, Provisorische Ärztevertretung, author's translation. Original text: ‘Aufgabe dieser gewählten Vertretung wird es sein, bei der künftigen Lösung des volksdeutschen Problems die besonderen Standesinteressen zu vertreten, entsprechende Vorschläge auszuarbeiten und bereits heute die Verbindung mit allen massgebenden Stellen aufzunehmen’.

97 OöLA, ZBST, Ordner 162–168, Sch.50, Denkschrift zur Lösung der volksdeutschen Ärztefrage, author's translation. Original text: ‘dass alle volksdeutschen Ärzte zur Betreuung ihrer Landsleute und zur Ausübung der ärztlichen Praxis in Österreich zugelassen werden und bezüglich ihrer Rechte und Pflichten den österreichischen Ärzten gleichgestellt werden’.

98 OöLA, ZBST, Ordner 162–168, Sch.50, Zentralberatungsstelle der VD. Landesstelle Salzburg.

99 *Salzburger Nachrichten*, 16 September 1949, 6.

100 OöLA, ZBST, Ordner 126–133, Sch. 45, Von der volksdeutschen fachärztlichen Beratungsstelle in Linz.

101 Bundesgesetzblatt für die Republik Österreich, Jahrgang 1952, 02.12.1952, 571, Bundesgesetz vom 15. Oktober 1952 über die Voraussetzungen der Ausübung der Rechtsanwaltschaft durch Volksdeutsche.

102 Cf. B. Scheuringer, ‘Die Situation der sudetendeutschen Flüchtlinge in Oberösterreich seit 1945’, *Mitteilungen des Oberösterreichischen Landesarchivs* 19 (2000), 141–76, 160.

103 Landsmann, ‘Politische Integration’, 69.

104 Ibid., 96.

105 Ther, ‘Integration of Expellees’, 791.

106 VLÖ Archiv, Box 10, SPOe-Mannöver um die Volksdeutschen.

107 M. Reiter and S. Crowe, ‘National Socialism in Austria before and after 1945: Nazi Minister Anton Reinthaller and the Origins of the Austrian Freedom Party’, *German Yearbook of Contemporary History* 5, 1 (2021), 120.

108 Wolff, ‘German Expellee Organizations’, 55.

109 Landsmann, ‘Politische Integration’, 70.

110 BÖ/O-zl. 9784-PB 257 bzw. z.Zahl 169.578 12U/1951, author's translation. Original text: ‘Von der Interessengemeinschaft der Volksdeutschen Heimatvertriebenen in Österreich wurde an uns das Ersuchen heran getragen, der Österreichische Gewerkschaftsbund sollte sich mit der völligen arbeitsrechtlichen Gleichstellung der volksdeutschen Arbeiter und Angestellten mit den österreichischen Arbeitnehmern und dem Wegfall der bisher vorgeschriebenen Befreiungsscheine einverstanden erklären. Ich bin nunmehr in der Lage, Ihnen, sehr geehrter Herr Bundesminister, mitteilen zu können, dass der Gewerkschaftsbund den Wunsch der Volksdeutschen für berechtigt hält und ersuche ihre Gleichstellung so bald als möglich in die Wege leiten zu wollen.’

111 Cf. Stenographisches Protokoll, 23. Sitzung des Nationalrates der Republik Österreich, 07.12.1953.

112 F. Frank and G. Wildmann, ‘Die Integration der Heimatvertriebenen deutscher Muttersprache’. Available at https://www.donauschwaben-ooe.at/geschichte/wissenschaftlich-betrachtet/bericht-19 (accessed 5 November 2024).

113 Bundesgesetz vom 2. Juni 1954, betreffend den Erwerb der Staatsbürgerschaft durch Volksdeutsche, https://www.ris.bka.gv.at/Dokumente/BgblPdf/1954_142_0/1954_142_0.pdf (accessed 5 November), author's translation. Original text: ‘Personen deutscher Sprachzugehörigkeit, die staatenlos sind oder deren Staatsangehörigkeit ungeklärt ist (Volksdeutsche), erwerben durch Erklärung, der österreichischen Republik als getreue Staatsbürger angehören zu wollen, die Staatsbürgerschaft’.

114 Fisher, ‘Ethnic Germans’, 154.

115 N. Kapeller, *Gedenkschrift: Verband der Volksdeutschen Landsmannschaften Österreichs* (Vienna 2014), 18.

116 Radspieler, ‘The Ethnic German Refugee’, 166.

